# Four New Fungal Species in Forest Ecological System from Southwestern China

**DOI:** 10.3390/jof10030194

**Published:** 2024-03-02

**Authors:** Yinglian Deng, Jinfa Li, Changlin Zhao, Jian Zhao

**Affiliations:** 1 College of Biodiversity Conservation, Southwest Forestry University, Kunming 650224, China; fungiyingliand@163.com; 2 Yingjiang Branch Bureau of Ecological Environment, Dehong 679300, China; yjhbstg@163.com; 3 Key Laboratory for Forest Resources Conservation and Utilization in the Southwest Mountains of China, Ministry of Education, Southwest Forestry University, Kunming 650224, China

**Keywords:** Asia, macrofungi, molecular systematics, taxonomy, Yunnan province

## Abstract

Four new wood-inhabiting fungi were found in Southwestern China within the genera *Phanerochaete*, *Phlebiopsis*, *Asterostroma*, and *Vararia* of the families Phanerochaetaceae and Peniophoraceae, belonging to the orders Polyporales and Russulales individually. Combined with their morphological characteristics and molecular biological evidence, the present study describes them as new fungal taxa. *Asterostroma yunnanense* is characterized by the resupinate, membranaceous to pellicular basidiomata with a cream to salmon-buff hymenial surface, hyphal system dimitic bearing simple-septa, thin- to thick-walled, yellowish brown asterosetae with acute tips, and thin-walled, echinulate, amyloid, globose basidiospores. *Phanerochaete tongbiguanensis* is characterized by the resupinate basidiomata with a white to cream hymenial surface, a monomitic hyphal system with simple-septa generative hyphae, the presence of subclavate cystidia covered with a lot of crystals, and oblong ellipsoid basidiospores (6–9 × 3–4.5 µm). *Phlebiopsis fissurata* is characterized by the membranaceous, tuberculate basidiomata with a buff to slightly brown hymenial surface, a monomitic hyphal system with simple-septa, conical cystidia, and broadly ellipsoid. *Vararia yingjiangensis* is characterized by a corky basidiomata with a pinkish buff to cinnamon-buff hymenial surface, cracking, yellowish dichohyphae with slightly curved tips, subulate gloeocystidia, and thick-walled, ellipsoid basidiospores (6.5–11.5 × 5–7 µm). The phylogenetic analyses of ITS + nLSU revealed that the two new species were nested into the genera *Phanerochaete* and *Phlebiopsis* within the family Phanerochaetaceae (Polyporales), in which *Phanerochaete tongbiguanensis* was sister to *P. daliensis*; *Phlebiopsis fissurata* was grouped with *P. lamprocystidiata*. Two new species were clustered into the genera *Asterostroma* and *Vararia* within the family Peniophoraceae (Russulales), in which *Asterostroma yunnanense* was sister to *A. cervicolor*; *Vararia yingjiangensis* formed a single branch.

## 1. Introduction

Over the past 30 years, wood-inhabiting basidiomycetes have been extensively studied in Chinese forests, and nearly 1600 species of wood-inhabiting basidiomycetes have been found in China [1,2,3,4,5,6,7,8,9,10]. One survey showed that 86 percent of species cause white rot, and 14 percent cause brown rot [11]. Two-order Polyporales Gäum. and Russulales Kreisel ex P.M. Kirk, P.F. Cannon, and J.C. David are diverse groups of the class Agaricomycetes Doweld (Basidiomycota R.T. Moore) [12].

The genus *Asterostroma* Massee belongs to the family Peniophoraceae Lotsy (Russulales, Basidiomycota), and it is typified with *Corticium apalum* Berk & Broome. It is characterized by the resupinate, membranaceous to pellicular basidiocarps, a dimitic (asterodimitic) hyphal system, simple-septate generative hyphae, dextrinoid asterosetae, the presence of gloeocystidia, and smooth or ornamented basidiospores with or without amyloid reactions [13,14,15,16]. Based on the MycoBank database (http://www.MycoBank.org, accessed on 22 February 2024) and the Index Fungorum (http://www.indexfungorum.org, accessed on 22 February 2024), 38 specific and infraspecific names have been registered in *Asterostroma*, but the actual number of the species has reached 31, and it is still poorly studied in China [17,18]. The wood-inhabiting fungal genus *Phanerochaete* P. Karst. belonged to the family Phanerochaetaceae Jülich (Polyporales, Basidiomycota), typified by *P. alnea* (Fr.) P. Karst. [19]. it is characterized by white-rot, resupinate, and membranaceous basidiocarps; a smooth or tuberculate hymenial surface; a monomitic hyphal system; generative hyphae mostly simple-septate; the presence of smooth or encrusted cystidia; and thin-walled, non-amyloid, and acyanophilous basidiospores [20,21,22,23]. Based on the MycoBank database (http://www.MycoBank.org, accessed on 22 February 2024) and the Index Fungorum (http://www.indexfungorum.org, accessed on 22 February 2024), the genus *Phanerochaete* has 210 specific and registered names, but the actual number of species has reached 112 [23,24,25,26,27,28]. The genus *Phlebiopsis* Jülich (Phanerochaetaceae, Polyporales), typified with *P. gigantea* (Fr.) Jülich, is characterized by a combination of resupinate to effused-reflexed basidiomata with a membranaceous to subceraceous consistency when fresh, cracked when dry, a smooth to odontoid to poroid hymenophore, a monomitic hyphal system with colorless, generative hyphae with simple-septate, hyaline cystidia that are thick-walled and encrusted, usually narrowly clavate basidia, and basidiospores that are hyaline, thin-walled, smooth, cylindrical to ellipsoid, acyanophilous, and negative in Melzer’s reagent [13,29]. So far, the MycoBank database (http://www.MycoBank.org, accessed on 22 February 2024) and Index Fungorum (http://www.indexfungorum.org, accessed on 22 February 2024) have registered 39 specific and infraspecific names for *Phlebiopsis*, but the actual number of the species has reached 33, and 6 species were transferred to *Phaeophlebiopsis* Floudas & Hibbett [26,30,31,32,33,34,35]. Recently, more than 150 specimens of the genus *Phlebiopsis* were collected by the mycologist from China and Southeast Asia [26,35]. The genus *Vararia* P. Karst. (Peniophoraceae, Russulales), typified by *V. investiens* (Schwein.) P. Karst., is a corticioid wood-inhabiting fungal genus with a wide distribution [13]. The genus is characterized by the resupinate basidiomata, a dimitic hyphal structure with simple-septate or clamped generative hyphae and often dextrinoid dichohyphae in Melzer’s reagent, the presence of gloeocystidia, and variously shaped smooth basidiospores with or without an amyloid reaction [13,36,37,38]. Based on the MycoBank database (http://www.MycoBank.org, accessed on 22 February 2024) and the Index Fungorum (http://www.indexfungorum.org, accessed on 22 February 2024), there are 99 specific and infraspecific names in *Vararia* [13,39,40,41,42,43,44]. But the actual number of species has reached 76, and they occur mainly in the tropical and subtropical areas of the world [8,42,43,44,45,46,47,48,49,50,51,52].

Pioneering research according to the family Phanerochaetaceae Jülich (Polyporales) and Peniophoraceae Lotsy (Russulales) was just the prelude to the molecular systematics of Basidiomycota [25,53,54,55]. Based on the nuclear rDNA ITS1-5.8S-ITS2 (ITS), the D1–D2 domains of 28S rDNA (28S), and the RNA polymerase II largest subunit (rpb1) genes, the phylogenetic diversity revealed that the taxa of Polyporales nested in the phlebioid clade, which included the family of Phanerochaetaceae, Irpicaceae Spirin & Zmitr., and Meruliaceae Rea, in which the result showed that 54 genera were included [12,25,54,55,56,57,58,59]. Species diversity, taxonomy, and multigene phylogeny revealed that the family Phanerochaetaceae comprises four main lineages with substantial support, including the *Donkia* Pilát, *Phanerochaete*, *Phlebiopsis*, and *Bjerkandera* P. Karst. Clades, in which *Phanerochaete* s.l. was defined as a polyphyletic genus based on previous phylogeny results [25]. Revisiting the taxonomy of *Phanerochaete* (Phanerochaetaceae, Polyporales) based on RPB1, RPB2, and the ITS and LSU revealed that *Phanerochaete* was further divided into four smaller clades (*Phanerochaete sensu stricto*, *Bjerkandera*, *Hyphodermella* J. Erikss. & Ryvarden, and *Phlebiopsis*); however, only *Phanerochaete* s.s. and *Phlebiopsis* clades have been previously identified [22]. The family Peniophoraceae (Russulales) was a large and rather heterogeneous family, although it appeared monophyletic in most analyses, and it was almost totally dominated by corticioid species, and the prime exception was the clavarioid genus *Lachnocladium* Lév. [53,60]. The phylogenetic diversity displayed by the corticioid fungal species based on 5.8S and 28S nuclear rDNA revealed that the taxa of Peniophoraceae were nested in the russuloid clade, which held a considerable share of the phylogenetic framework [14,15,16,61]. The phylogenetic research about the major clades of mushroom-forming fungi (Homobasidiomycetes) indicate that the largest resupinate forms were divided into the polyporoid clade, russuloid clade, and hymenochaetoid clade, in which *Peniophora* Cooke was grouped with *Asterostroma* and *Scytinostroma* Donk [54]. Re-thinking the classification of corticioid fungi to clear the phylogenetic relationships inferred from 5.8S and nLSU rDNA sequences using Bayesian analysis showed that *Asterostroma*, *Gloiothele* Bres., *Peniophora*, *Scytinostroma*, and *Vararia* were clustered in the family Peniophoraceae (Russulales) [41,53].

During the investigations on wood-inhabiting fungi in Yunnan province, China, four new species were found, which could not be assigned to any described species. We present the morphological and molecular phylogenetic evidence that support the recognition of these four new species in Phanerochaetaceae and Peniophoraceae based on the internal transcribed spacer (ITS) regions and the large subunit nuclear ribosomal RNA gene (nLSU) sequences.

## 2. Materials and Methods

### 2.1. Sample Collection and Herbarium Specimen Preparation

Fresh fruiting bodies of basidiomycetous macrofungi were collected from Lincang, Dehong, Yunnan province, P.R. China. Specimens were dried in an electric food dehydrator at 40 °C and then sealed and stored in an envelope bag and deposited in the herbarium of Southwest Forestry University (SWFC), Kunming, Yunnan province, P.R. China. Macromorphological descriptions are based on field notes and photos captured in the field and lab.

### 2.2. Molecular Phylogeny

Macromorphological descriptions and color terminology are based on field notes and photos captured in the field or lab, and they follow those of a previous study [54]. The micromorphological data were obtained from the dried specimens based on observing them under a light microscope following a previous study [55]. The following abbreviations are used: KOH = 5% potassium hydroxide water solution, CB = Cotton Blue, CB– = acyanophilous, IKI = Melzer’s reagent, IKI– = both inamyloid and indextrinoid, L = mean spore length (arithmetic average for all spores), W = mean spore width (arithmetic average for all spores), Q = variation in the L/W ratios between the specimens studied, and n = a/b (number of spores (a) measured from given number (b) of specimens).

### 2.3. DNA Extraction and Sequencing

According to the manufacturer’s instructions, we used the CTAB rapid plant genome extraction kit-DN14 (Aidlab Biotechnologies Co., Ltd., Kunming, China) to obtain genomic DNA from dried specimens [62]. A total of 3 µL of DNA was evenly mixed with 3 µL 5 × bromophenol blue indicator and a 3 µL DNA sample to be tested, and the samples were placed on a 1.5% agarose gel plate (containing 0.5 µg/mL EB). The DNA molecular weight was labeled DL 2000 with a molecular weight of 560–23,130 bp, and the pressure was stabilized at 90 V. Electrophoresis occurred for 30 min. The nuclear ribosomal ITS region was amplified with primers ITS5 (GGA AGT AAA AGT CGT AAC AAG G) and ITS4 (TCC TCC GCT TAT TGA TAT GC) [62]. The nuclear nLSU region was amplified with primer pair LR0R (ACC CGC TGA ACT TAA GC) and LR7 (TAC TAC CAC CAA GAT CT) [62]. The basic amplification reaction system of ITS and nLSU is shown in Table 1. And the newly generated sequences were deposited in NCBI GenBank (Table 2).

### 2.4. Phylogenetic Analyses

**Figure 1:**
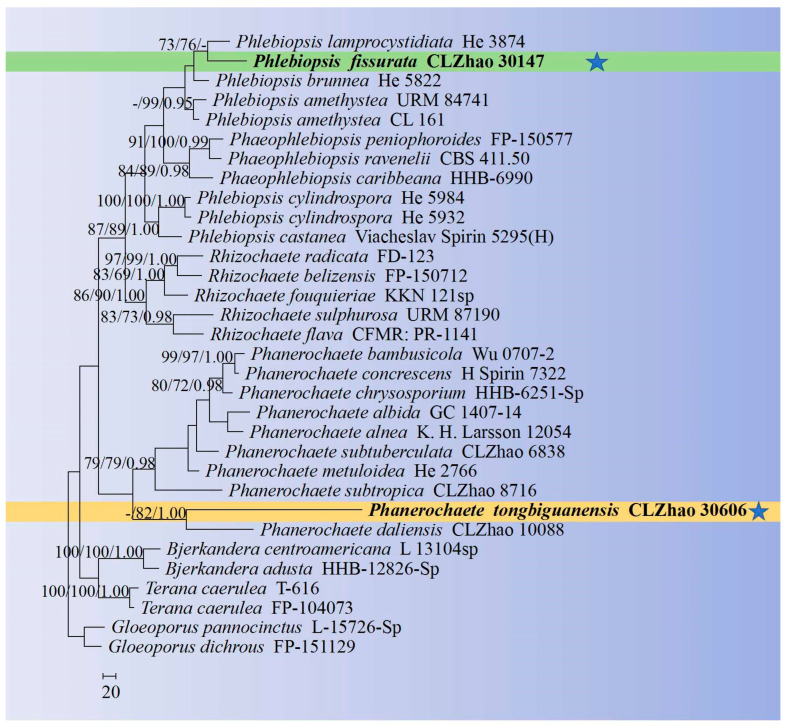
Maximum parsimony is a strict consensus tree illustrating the phylogeny of two new species and related genera in the family Phanerochaetaceae based on ITS + nLSU sequences. The new species are marked with asterisks.

**Figure 2:**
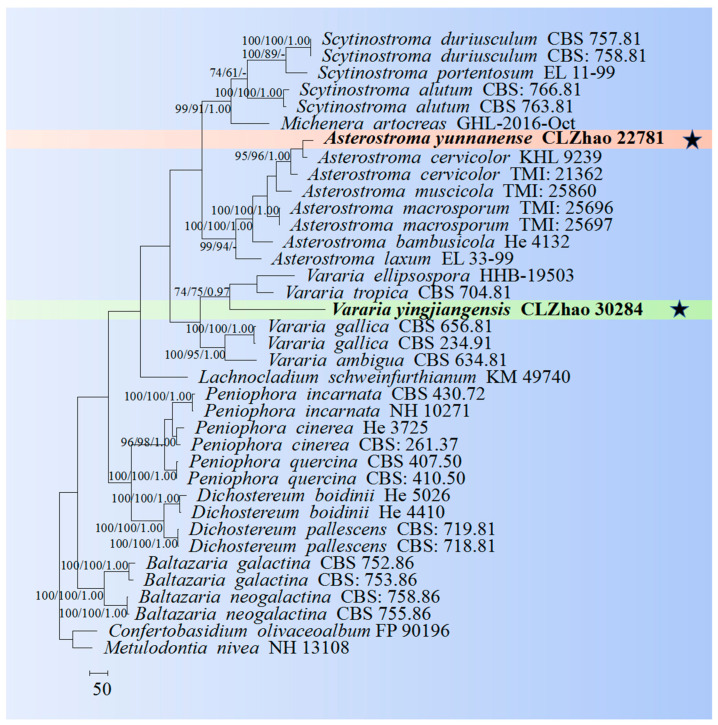
Maximum parsimony is a strict consensus tree illustrating the phylogeny of two new species and related genera in the family Peniophoraceae based on ITS + nLSU sequences. The new species are marked with asterisks.

**Figure 3:**
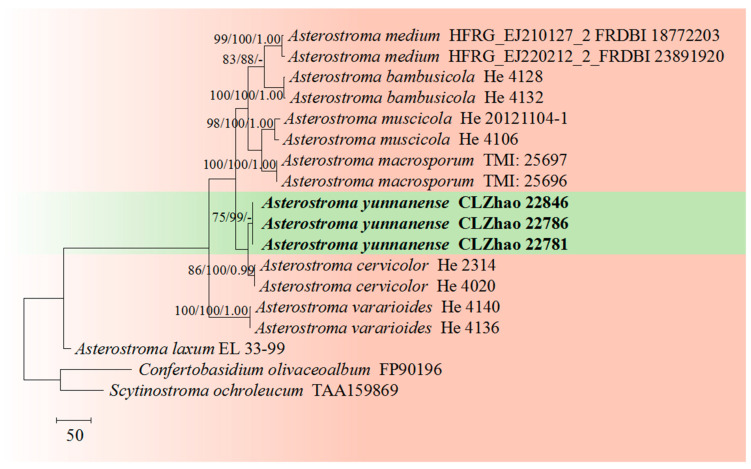
Maximum parsimony is a strict consensus tree illustrating the phylogeny of the new species and related species in the genus *Asterostroma* based on ITS sequences.

**Figure 4:**
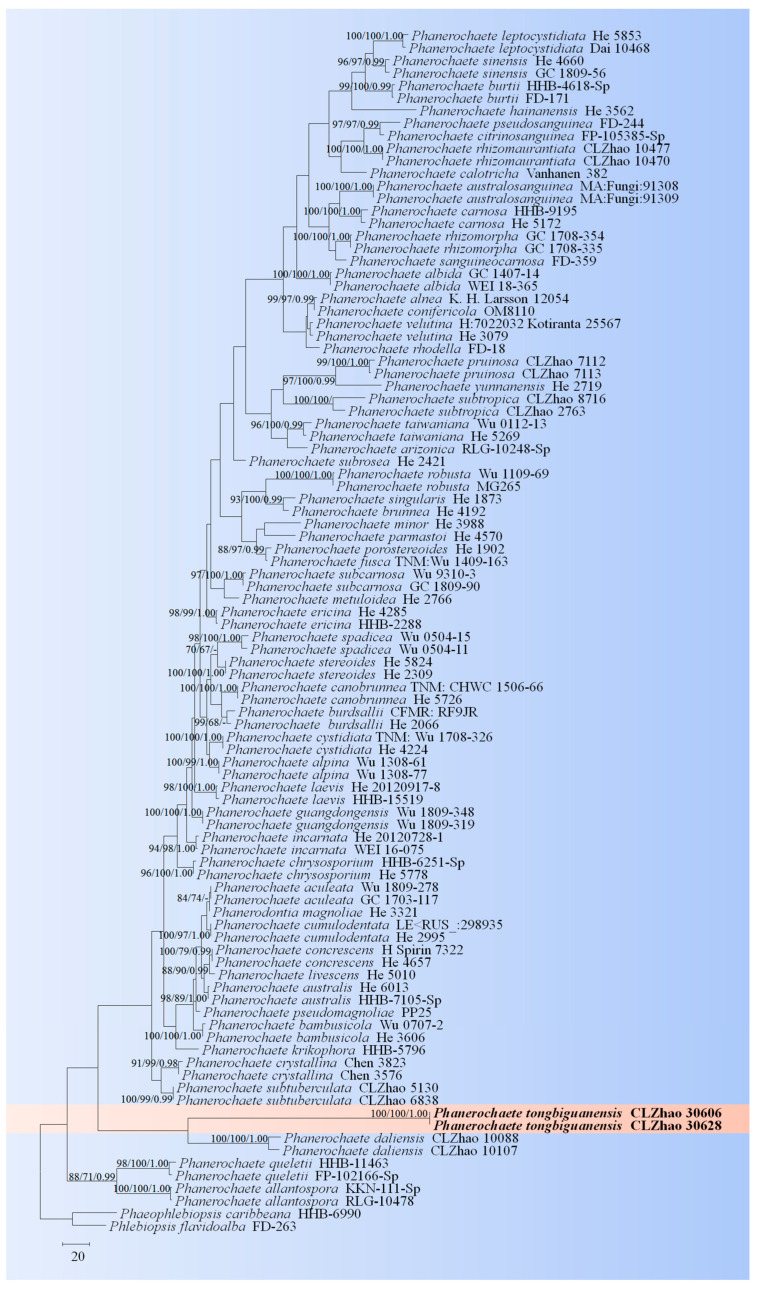
Maximum parsimony is a strict consensus tree illustrating the phylogeny of the new species and related species in the genus *Phanerochaete* based on ITS sequences.

**Figure 5:**
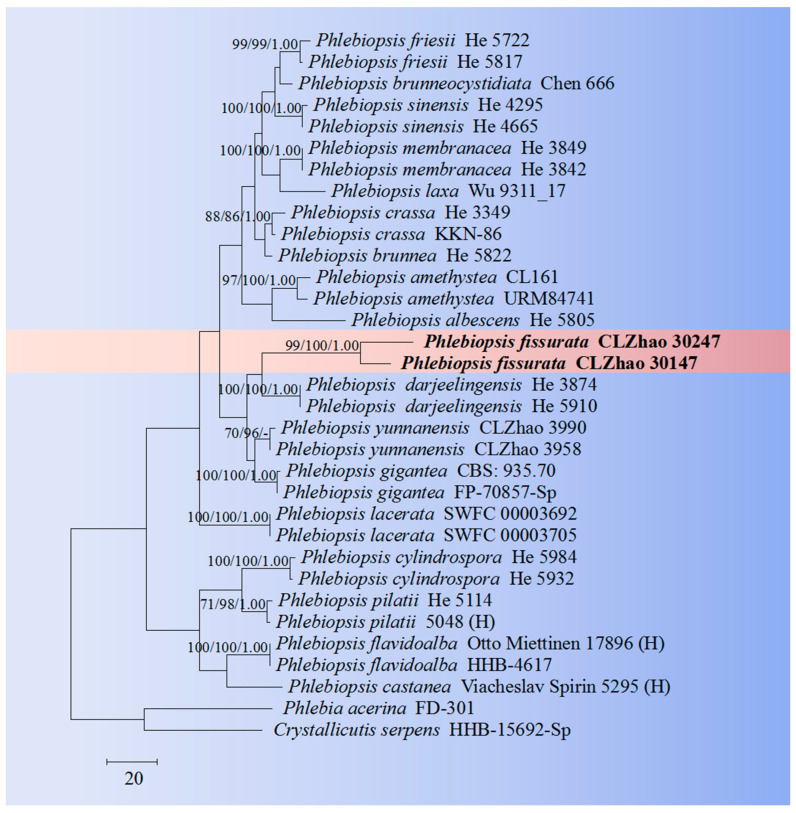
Maximum parsimony is a strict consensus tree illustrating the phylogeny of the new species and related species in the genus *Phlebiopsis* based on ITS sequences.

**Figure 6:**
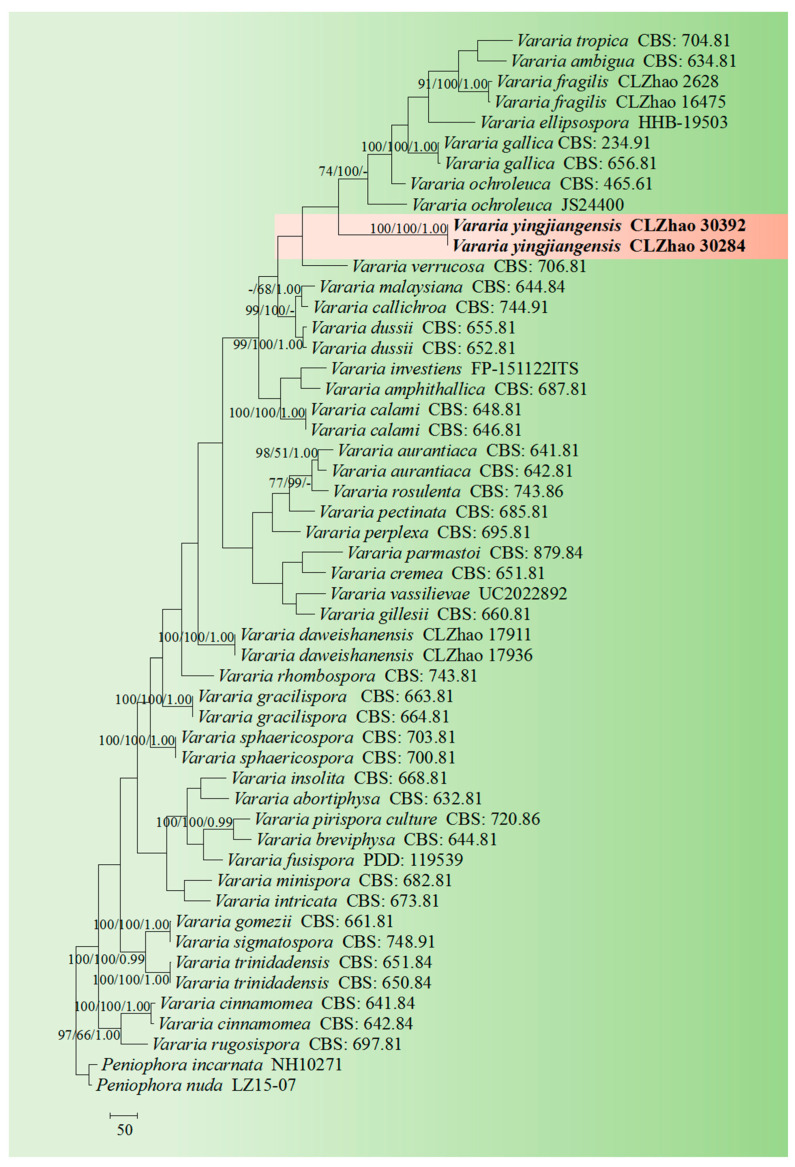
Maximum parsimony is a strict consensus tree illustrating the phylogeny of the new species and related species in the genus *Vararia* based on ITS sequences.

The sequences were aligned in MAFFT 7 (https://mafft.cbrc.jp/alignment/server/, 20 December 2023) using the “G-INS-i” strategy for the ITS and nLSU datasets and manually adjusted in BioEdit [63]. Sequences of *Gloeoporus pannocinctus* (Romell) J. Erikss. and *G. dichrous* (Fr.) Bres. Obtained from GenBank were selected as an outgroup for phylogenetic analysis of the ITS + nLSU phylogenetic tree (Figure 1) [64]. Sequences of *Confertobasidium olivaceoalbum* (Bourdot & Galzin) Jülich and *Metulodontia nive* (P. Karst.) Parmasto retrieved from GenBank were used as outgroups in the ITS + nLSU (Figure 2) analysis following a previous study [65]. The sequences of *Phaeophlebiopsis caribbeana* Floudas & Hibbett and *Phlebiopsis flavidoalba* (Cooke) Hjortstam were selected as an outgroup in the ITS analysis (Figure 3) following a previous study [64]. The sequences of *Crystallicutis serpens* (Tode) El-Gharabawy, Leal-Dutra & G.W. Griff., and *Phlebia acerina* Peck were selected as an outgroup for the phylogenetic analysis of ITS phylogenetic tree (Figure 4) [29]. The sequences of *Confertobasidium olivaceoalbum* (Bourdot & Galzin) Jülich and *Scytinostroma ochroleucum* Donk were selected as an outgroup for the phylogenetic analysis of ITS phylogenetic tree (Figure 5) [35]. The sequences of *Peniophora incarnata* (Pers.) P. Karst. and *Peniophora nuda* (Fr.) Bres. retrieved from GenBank were used as outgroups in the ITS (Figure 6) analysis following a previous study [65].

Maximum parsimony (MP), maximum likelihood (ML), and Bayesian inference (BI) analyses were applied to the combined three datasets [66]. BS (Branch Support) for ML (maximum likelihood) analysis was determined by 1000 bootstrap replicates, and bootstrap values were >70% [66]. MP (maximum parsimony) analysis was performed in PAUP* version 4.0b10, and parsimony bootstrap values were >50% [67]. BI (Bayesian inference) and clade robustness were assessed using bootstrap (BT) analysis with 1000 replicates, and Bayesian posterior probabilities were >0.95 [68,69]. All of the characters were equally weighted, and gaps were treated as missing data. Trees were inferred using the heuristic search option with TBR branch swapping and 1000 random sequence additions. Max trees were set to 5000, branches of zero length were collapsed, and all parsimonious trees were saved. Clade robustness was assessed using bootstrap (BT) analysis with 1000 replicates [68]. Descriptive tree statistics, tree length (TL), consistency index (CI), retention index (RI), rescaled consistency index (RC), and homoplasy index (HI) were calculated for each maximum parsimonious tree generated. The multiple sequence alignment was also analyzed using maximum likelihood (ML) in RAxML-HPC2 through the Cipres Science Gateway [69].

**Table 2:** List of species, specimens, and GenBank accession numbers of sequences used in this study. The new species are in bold.

Species Name	Specimen No.	GenBank Accession No.		Country	References
		ITS	nLSU		
*Asterostroma bambusicola*	He4132	KY263865	KY263871	China	[42]
*A. bambusicola*	He4128	KY263864		China	[42]
*A. cervicolor*	He4020	KY263860	KY263868	China	[42]
*A. cervicolor*	He2314	KY263859	KY263869	China	[42]
*A. cervicolor*	TMI:21362	AB439560		Japan	[17]
*A. cervicolor*	KHL9239	AF506408	AF506408	Sweden	[41]
*A. laxum*	EL33-99	AF506410	AF506410	Sweden	[41]
*A. macrosporum*	TMI:25696	AB439544		Japan	[17]
*A. macrosporum*	TMI:25697	AB439545		Japan	[17]
*A. medium*	HFRG_EJ220212_2_FRDBI 23891920	OQ133615		United Kingdom	Unpublished
*A. medium*	HFRG_EJ210127_2 FRDBI 18772203	OL828779		United Kingdom	Unpublished
*A. muscicola*	He20121104-1	KY263862	KY263872	China	[42]
*A. muscicola*	He4106	KY263861	KY263873	China	[42]
*A. muscicola*	TMI:25860	AB439551		Japan	[17]
*A. vararioides*	He4140	KY263867	KY263870	China	[42]
*A. vararioides*	He4136	KY263866		China	[42]
***A. yunnanense***	CLZhao 22781 *	**OR048809**	**OR506285**	**China**	**Present study**
***A. yunnanense***	**CLZhao 22846**	**OR048810**	**OR506287**	**China**	**Present study**
***A. yunnanense***	**CLZhao 22786**	**OR048811**	**OR506286**	**China**	**Present study**
*Baltazaria galactina*	CBS 752.86	MH862034	MH873721	France	[70]
*B. galactina*	CBS:753.86	MH862035	MH873722	France	[70]
*B. neogalactina*	CBS 755.86	MH862037	MH873724	France	[70]
*B. neogalactina*	CBS:758.86	MH862040	MH873727	France	[70]
*Bjerkandera adusta*	HHB-12826-Sp	KP134983	KP135198	USA	[22]
*B. centroamericana*	L13104sp	KY948791	KY948855	Costa Rica	[56]
*Confertobasidium olivaceoalbum*	FP90196	AF511648	AF511648	Sweden	[41]
*Crystallicutis serpens*	HHB-15692-Sp	KP135031	KP135200	USA	[22]
*Dichostereum boidinii*	He4410	MH538315	MH538331	China	[70]
*D. boidinii*	He5026	MH538324	MH538330	China	[71]
*D. pallescens*	CBS:718.81	MH861456	MH873198	USA	[70]
*D. pallescens*	CBS:719.81	MH861457	MH873199	USA	[70]
*Gloeoporus dichrous*	FP-151129	KP135058		USA	[22]
*G. pannocinctus*	L-15726-Sp	KP135060		USA	[22]
*Lachnocladium schweinfurthianum*	KM49740	MH260033	MH260051	United Kingdom	[65]
*Metulodontia nivea*	NH13108	AF506423	AF506423	Sweden	[41]
*M. artocreas*	GHL-2016-Oct	MH204688	MH204692	USA	[72]
*Peniophora cinerea*	He3725	MK588769	MK588809	China	Unpublished
*P. cinerea*	CBS:261.37	MH855905	MH867412	Belgium	[70]
*P. incarnata*	CBS 430.72	MH860518	MH872230	Netherlands	[70]
*P. incarnata*	NH10271	AF506425	AF506425	Sweden	[41]
*P. quercina*	CBS 407.50	MH856687	MH868204	France	[70]
*P. quercina*	CBS:410.50	MH856690	MH868207	France	[70]
*Phaeophlebiopsis caribbeana*	HHB-6990	KP135415	KP135243	USA	[22]
*P. peniophoroides*	FP-150577	KP135417	KP135273	USA	[22]
*P. ravenelii*	CBS:411.50	MH856691	MH868208	France	[70]
*Phanerochaete burdsallii*	He 2066	MT235690	MT248177	USA	Unpublished
*P. aculeata*	Wu 1809-278	MZ422786	MZ637178	China	[25]
*P. aculeata*	GC 1703-117	MZ422785	MZ63717	China	[25]
*P. albida*	WEI 18-365	MZ422789	MZ637180	China	[25]
*P. albida*	GC 1407-14	MZ422788	MZ637179	China	[25]
*P. allantospora*	KKN-111-Sp	KP135038	KP135238	USA	[22]
*P. allantospora*	RLG-10478	KP135039		USA	[22]
*P. alnea*	K. H. Larsson 12054	KX538924		Norway	[73]
*P. alnea voucher*	K. H. Larsson 12054	KX538924		Norway	[73]
*P. alpina*	Wu 1308-61	MZ422790	MZ637182	China	[25]
*P. alpina*	Wu 1308-77	MZ422791	MZ637183	China	[25]
*P. arizonica*	RLG-10248-Sp	KP135170	KP135239	USA	[22]
*P. australis*	He 6013	MT235656	MT248136	China	[74]
*P. australis*	HHB-7105-Sp	KP135081	KP135240	USA	[22]
*P. australosanguinea*	MA:Fungi:91308	MH233925	MH233928	Chile	[74]
*P. australosanguinea*	MA:Fungi:91309	MH233926	MH233929	Chile	[74]
*P. bambusicola*	He 3606	MT235657	MT248137	China	[25]
*P. bambusicola*	Wu 0707-2	MF399404	MF399395	China	[75]
*P. brunnea*	He 4192	MT235658	MT248138	China	[76]
*P. burdsallii*	CFMR:RF9JR	KU668973		USA	[27]
*P. burtii*	HHB-4618-Sp	KP135117	KP135241	USA	[22]
*P. burtii*	FD-171	KP135116		USA	[22]
*P. calotricha*	Vanhanen382	KP135107		USA	[22]
*P. canobrunnea*	He 5726	MT235659	MT248139	Sri Lanka	[75]
*P. canobrunnea*	TNM:CHWC 1506-66	LC412095	LC412104	China	[76]
*P. carnosa*	He 5172	MT235660	MT248140	China	[76]
*P. carnosa*	HHB-9195	KP135129	KP135242	USA	[22]
*P. chrysosporium*	He 5778	MT235661	MT248141	Sri Lanka	[76]
*P. chrysosporium*	HHB-6251-Sp	KP135094	KP135246	USA	[22]
*P. citrinosanguinea*	FP-105385-Sp	KP135100	KP135234	USA	[22]
*P. concrescens*	He 4657	MT235662	MT248142	China	[25]
*P. concrescens*	H Spirin 7322	KP994380	KP994382	Russia	[77]
*P. conifericola*	OM8110	KP135171		Finland	[22]
*P. crystallina*	Chen 3823	MZ422802	MZ637188	China	[25]
*P. crystallina*	Chen 3576	MZ422801		China	[25]
*P. cumulodentata*	He 2995	MT235664	MT248144	China	[74]
*P. cumulodentata*	LE < RUS_:298935	KP994359	KP994386	Russia	[77]
*P. cystidiata*	He 4224	MT235665	MT248145	China	[76]
*P. cystidiata*	TNM:Wu 1708-326	LC412097	LC412100	China	[78]
*P. daliensis*	CLZhao F10107	OP605506	OP874696	China	[27]
*P. daliensis*	CLZhao F10088	OP605505	OP874695	China	[27]
*P. ericina*	HHB-2288	KP135167	KP135247	USA	[22]
*P. ericina*	He 4285	MT235666	MT248146	China	[76]
*P. fusca*	TNM:Wu 1409-163	LC412099	LC412106	China	[78]
*P. guangdongensis*	Wu 1809-348	MZ422813	MZ637199	China	[25]
*P. guangdongensis*	Wu 1809-319	MZ422811	MZ637197	China	[25]
*P. hainanensis*	He 3562	MT235692	MT248179	China	[24]
*P. incarnata*	He 20120728-1	MT235669	MT248149	China	[76]
*P. incarnata*	WEI 16-075	MF399406	MF399397	China	[75]
*P. krikophora*	HHB-5796	KP135164	KP135268	USA	[22]
*P. laevis*	He 20120917-8	MT235670	MT248150	China	[76]
*P. laevis*	HHB-15519	KP135149	KP135249	USA	[22]
*P. leptocystidiata*	He 5853	MT235685	MT248168	China	[76]
*P. leptocystidiata*	Dai 10468	MT235684	MT248167	China	[76]
*P. livescens*	He 5010	MT235671	MT248151	China	[76]
*P. metuloidea*	He 2766	MT235682	MT248164	China	[76]
*P. minor*	He 3988	MT235686	MT248170	China	[76]
*P. parmastoi*	He 4570	MT235673	MT248153	China	[76]
*P. porostereoides*	He1902	KX212217	KX212221	China	[42]
*P. pruinosa*	CLZhao 7112	MZ435346	MZ435350	China	[64]
*P. pruinosa*	CLZhao 7113	MZ435347	MZ435351	China	[64]
*P. pseudomagnoliae*	PP25	KP135091	KP135250	South Africa	[22]
*P. pseudosanguinea*	FD-244	KP135098	KP135251	USA	[22]
*P. queletii*	FP-102166-Sp	KP134995		USA	[22]
*P. queletii*	HHB-11463	KP134994	KP135235	USA	[22]
*P. rhizomaurantiata*	CLZhao 10470	MZ435348	MZ435352	China	[64]
*P. rhizomaurantiata*	CLZhao 10477	MZ435349	MZ435353	China	[64]
*P. rhizomorpha*	GC 1708-335	MZ422824	MZ637208	China	[24]
*P. rhizomorpha*	GC 1708-354	MZ422825	MZ637209	China	[25]
*P. rhodella*	FD-18	KP135187	KP135258	USA	[22]
*P. robusta*	Wu 1109-69	MF399409	MF399400	China	[78]
*P. robusta*	MG265	KP127068	KP127069	China	[23]
*P. sanguineocarnosa*	FD-359	KP135122	KP135245	USA	[22]
*P. sinensis*	He 4660	MT235688	MT248175	China	[76]
*P. sinensis*	GC 1809-56	MT235689	MT248176	China	[76]
*P. singularis*	He1873	KX212220	KX212224	China	[78]
*P. spadicea*	Wu 0504-15	MZ422837	MZ637219	China	[25]
*P. spadicea*	Wu 0504-11	MZ422836		China	[25]
*P. stereoides*	He 5824	MT235677	MT248158	Sri Lanka	[76]
*P. stereoides*	He2309	KX212219	KX212223	China	[42]
*P. subcarnosa*	Wu 9310-3	MZ422841	GQ470642	China	[21]
*P. subcarnosa*	GC 1809-90	MZ422840	MZ637222	China	[25]
*P. subrosea*	He 2421	MT235687	MT248174	China	[76]
*P. subtropica*	CLZhao F8716	OP605486	OQ195089	China	[27]
*P. subtropica*	CLZhao F2763	OP605518	OQ195090	China	[27]
*P. subtropica*	CLZhao F8716	OP605486	OQ195089	China	[27]
*P. subtuberculata*	CLZhao F5130	OP605484	OQ195088	China	[27]
*P. subtuberculata*	CLZhao F6838	OP605485	OQ195087	China	[27]
*P. subtuberculata*	CLZhao F6838	OP605485	OQ195087	China	[27]
*P. taiwaniana*	He 5269	MT235680	MT248161	Vietnam	[76]
*P. taiwaniana*	Wu 0112-13	MF399412	MF399403	China	[75]
***P. tongbiguanensis***	CLZhao 30606 *	**OR917875**	**OR921222**	**China**	**Present study**
***P. tongbiguanensis***	**CLZhao 30628**	**OR917876**		**China**	**Present study**
*P. velutina*	He 3079	MT235681	MT248162	China	[76]
*P. velutina*	H:7022032 Kotiranta 25567	KP994354	KP994387	Russia	[77]
*P. yunnanensis*	He 2719	MT235683	MT248166	China	[76]
*Phanerodontia magnoliae*	He 3321	MT235672	MT248152	China	[76]
*Phlebiopsis albescens*	He 5805	MT452526		China	[35]
*P. amethystea*	CL161	MK993644	MK993638	Brazil	[79]
*P. amethystea*	URM84741	MK993645	MK993639	China	[66]
*P. brunnea*	He 5822	MT452527	MT447451	China	[35]
*P. brunneocystidiata*	Chen 666	MT561707	GQ470640	China	[21]
*P. castanea*	Viacheslav Spirin 5295 (H)	KX752610	KX752610	Russia	[80]
*P. crassa*	He 3349	MT561712	MT447407	China	[35]
*P. crassa*	KKN-86	KP135394	KP135215	USA	[22]
*P. cylindrospora*	He5932	MT386403	MT447444	China	[35]
*P. cylindrospora*	He5984	MT386404	MT447445	China	[35]
*P. lamprocystidiata*	He5910	MT386383	MT386383	China	[35]
*P. lamprocystidiata*	He3874	MT386382	MT447418	China	[35]
***P. fissurata***	**CLZhao 30247**	**OR917878**	**OR921226**	**China**	**Present study**
***P. fissurata***	CLZhao 30147 *	**OR917877**	**OR921223**	**China**	**Present study**
*P. flavidoalba*	Otto Miettinen 17896 (H)	KX752607	KX752607	USA	[80]
*P. flavidoalba*	HHB-4617	KP135401	KP135401	USA	[22]
*P. flavidoalba*	FD-263	KP135402	KP135271	USA	[22]
*P. friesii*	He 5722	MT452528	MT447413	Sri Lanka	[35]
*P. friesii*	He 5817	MT452529	MT447414	Sri Lanka	[35]
*P. gigantea*	CBS:935.70	MH860011	MH871798	Germany	[70]
*P. gigantea*	FP-70857-Sp	KP135390	KP135272	USA	[22]
*P. lacerata*	SWFC00003692	MT180946	MT180950	China	Unpublished
*P. lacerata*	SWFC00003705	MT180947	MT180951	China	Unpublished
*P. laxa*	Wu 9311_17	MT561710	GQ470649	China	[21]
*P. membranacea*	He3842	MT386400		China	[35]
*P. membranacea*	He3849	MT386401		China	[35]
*P. pilatii*	He5114	MT386385		China	[35]
*P. pilatii*	Viacheslav Spirin 5048 (H)	KX752590	KX752590	Russia	[80]
*P. sinensis*	He4295	MT386395		China	[35]
*P. sinensis*	He4665	MT386396		China	[35]
*P. yunnanensis*	CLZhao 3958	MH744140	MH744142	China	[81]
*P. yunnanensis*	CLZhao 3990	MH744141	MH744143	China	[81]
*Rhizochaete belizensis*	FP-150712	KP135408	KP135280	Belize	[22]
*R. flava*	CFMR:PR-1141	KY273030	KY273033	Puerto Rico	[82]
*R. fouquieriae*	KKN121sp	KY948786	KY948858	USA	[57]
*R. radicata*	FD-123	KP135407	KP135279	USA	[22]
*R. sulphurosa*	URM87190	KT003522	KT003519	Brazil	[83]
*Scytinostroma alutum*	CBS:766.81	MH861486	MH873225	France	[70]
*S. alutum*	CBS 763.81	MH861483	MH873222	France	[70]
*S. duriusculum*	CBS 757.81	MH861477	MH873216	France	[70]
*S. duriusculum*	CBS:758.81	MH861478	MH873217	France	[70]
*S. ochroleucum*	TAA159869	AF506468	AF506468	Sweden	[41]
*S. portentosum*	EL11-99	AF506470	AF506470	Sweden	[41]
*Terana caerulea*	FP-104073	KP134980	KP135276	USA	[22]
*T. caerulea*	T-616	KP135276		USA	[22]
*Vararia abortiphysa*	CBS:632.81	MH861387	MH861387	Gabon	[70]
*V. ambigua*	CBS 634.81	MH861388	MH873137	France	[70]
*V. amphithallica*	CBS:687.81	MH861431	MH861431	France	[70]
*V. aurantiaca*	CBS:642.81	MH861394	MH861394	Gabon	[70]
*V. aurantiaca*	CBS:641.81	MH861393	MH861393	France	[70]
*V. breviphysa*	CBS:644.81	MH861396	MH861396	Gabon	[70]
*V. calami*	CBS:646.81	MH861398	MH861398	France	[70]
*V. calami*	CBS:648.81	MH861399	MH861399	France	[70]
*V. callichroa*	CBS:744.91	MH874000	MH874000	France	[70]
*V. cinnamomea*	CBS:642.84	MH873488	MH873488	Madagascar	[70]
*V. cinnamomea*	CBS:641.84	MH861794	MH861794	Madagascar	[70]
*V. cremea*	CBS:651.81	MH873147	MH873147	France	[70]
*V. daweishanensis*	CLZhao 17911	OP380613	OP615103	China	[43]
*V. daweishanensis*	CLZhao 17936	OP380614	OP380688	China	[43]
*V. dussii*	CBS:655.81	MH861405	MH861405	France	[70]
*V. dussii*	CBS:652.81	MH873148	MH873148	France	[70]
*V. ellipsospora*	HHB-19503	MW740328	MW740328	New Zealand	[43]
*V. fragilis*	CLZhao 2628	OP380611		China	[43]
*V. fragilis*	CLZhao 16475	OP380612	OP380687	China	[43]
*V. fusispora*	PDD:119539	OL709443	OL709443	New Zealand	[43]
*V. gallica*	CBS 234.91	MH862250	MH873932	Canada	[70]
*V. gallica*	CBS 656.81	MH861406	MH873152	France	[70]
*V. gillesii*	CBS:660.81	MH873153	MH873153	Cote d’Ivoire	[70]
*V. gomezii*	CBS:661.81	MH873154	MH873154	France	[70]
*V. gracilispora*	CBS:664.81	MH861412	MH861412	Gabon	[70]
*V. gracilispora*	CBS:663.81	MH861411		Gabon	[70]
*V. insolita*	CBS:668.81	MH861413	MH861413	France	[70]
*V. intricata*	CBS:673.81	MH861418	MH861418	France	[70]
*V. investiens*	FP-151122ITS	MH971976	MH971977	USA	[72]
*V. malaysiana*	CBS:644.84	MH873490	MH873490	Singapore	[70]
*V. minispora*	CBS:682.81	MH861426	MH861426	France	[70]
*V. ochroleuca*	CBS:465.61	MH858109	MH858109	France	[70]
*V. ochroleuca*	JS24400	AF506485	AF506485	Norway	[41]
*V. parmastoi*	CBS:879.84	MH861852	MH861852	Uzbekistan	[70]
*V. pectinata*	CBS:685.81	MH861429		Cote d’Ivoire	[70]
*V. perplexa*	CBS:695.81	MH861438	MH861438	France	[70]
*V. pirispora*	CBS:720.86	MH862016	MH862016	France	[70]
*V. rhombospora*	CBS:743.81	MH861470	MH861470	France	[70]
*V. rosulenta*	CBS:743.86	MH862028		France	[70]
*V. rugosispora*	CBS:697.81	MH861440	MH861440	Gabon	[70]
*V. sigmatospora*	CBS:748.91	MH874001	MH874001	Netherlands	[70]
*V. sphaericospora*	CBS:700.81	MH873185	MH873185	Gabon	[70]
*V. sphaericospora*	CBS:703.81	MH861446	MH861446	Gabon	[70]
*V. trinidadensis*	CBS:651.84	MH861803	MH861803	Madagascar	[70]
*V. trinidadensis*	CBS:650.84	MH873495	MH873495	Madagascar	[70]
*V. tropica*	CBS 704.81	MH861447	MH873189	France	[70]
*V. vassilievae*	UC2022892	KP814203	KP814203	USA	Unpublished
*V. verrucosa*	CBS:706.81	MH861449	MH861449	France	[70]
***V. yingjiangensis***	CLZhao 30284 *	**OR917879**	**OR921225**	**China**	**Present study**
***V. yingjiangensis***	**CLZhao 30392**	**OR917880**	**OR921224**	**China**	**Present study**

* Is shown in holotype.

The best-fit evolution model for each dataset for BI (Bayesian inference) was determined by using MrModeltest 2.3 [84]. BI was calculated with MrBayes3.1.2 with a general time reversible (GTR + I + G) model of DNA substitution and a gamma distribution rate variation rate variation across sites [85]. A total of four Markov chains were run for two runs from random starting trees for 2 million and 0.5 million generations for ITS + nLSU (Figure 1 and Figure 2), respectively, and based on ITS for 5 million generations (Figure 3), 0.5 million generations (Figure 4), for 0.5 million generations (Figure 5), and 0.2 million generations (Figure 6), with trees and parameters sampled every 1000 generations.

## 3. Results

### 3.1. Molecular Phylogeny

The ITS + nLSU dataset (Figure 1) included sequences from 32 fungal specimens representing 29 species. The dataset had an aligned length of 2550 characters, of which 1682 characters are constant, 424 are variable and parsimony-uninformative, and 444 are parsimony-informative. Maximum parsimony analysis yielded one equally parsimonious tree (TL = 2330, CI = 0.5408, HI = 0.4592, RI = 0.4861, RC = 0.2629). The best model for the ITS + nLSU dataset estimated and applied in the Bayesian analysis was GTR + I + G (lset nst = 6, rates = invgamma; prset statefreqpr = dirichlet (1,1,1,1). Bayesian analysis and ML analysis resulted in a similar topology to MP analysis with an average standard deviation of split frequencies = 0.008198 (BI), and the effective sample size (ESS) across the two runs is the double of the average ESS (avg ESS) = 1887. The phylogeny (Figure 1) based on the combined nLSU sequences includes six genera within the family Peniophoraceae: *Bjerkandera*, *Phaeophlebiopsis*, *Phanerochaete*, *Phlebiopsis*, *Rhizochaete* Gresl. and Nakasone & Rajchenb. and *Terana* Adans. Our current two new species were clustered into genera *Phanerochaete* and *Phlebiopsis*.

The ITS + nLSU dataset (Figure 2) included sequences from 37 fungal specimens representing 25 species. The dataset had an aligned length of 2573 characters, of which 1433 characters are constant, 383 are variable and parsimony-uninformative, and 757 are parsimony-informative. Maximum parsimony analysis yielded one equally parsimonious tree (TL = 3484, CI = 0.5347, HI = 0.4653, RI = 0.6921, RC = 0.3701). The best model for the ITS + nLSU dataset estimated and applied in the Bayesian analysis was GTR + I + G (lset nst = 6, rates = invgamma; prset statefreqpr = dirichlet (1,1,1,1). Bayesian analysis and ML analysis resulted in a similar topology to MP analysis with an average standard deviation of split frequencies = 0.005232 (BI), and the effective sample size (ESS) across the two runs is the double of the average ESS (avg ESS) = 304. The phylogeny (Figure 2) based on the combined ITS + nLSU sequences includes eight genera within the family Peniophoraceae: *Asterostroma*, *Baltazaria* Leal-Dutra, Dentinger & G.W. Griff., *Dichostereum* Pilát, *Lachnocladium*, *Michenera* Berk. & M.A. Curtis, *Peniophora*, *Scytinostroma*, and *Vararia*. Our current two new species were clustered into genera *Asterostroma* and *Vararia*.

The ITS dataset of the genus *Asterostroma* (Figure 3) included sequences from 18 fungal specimens representing 10 species. The dataset had an aligned length of 1560 characters, of which 983 characters are constant, 246 are variable and parsimony-uninformative, and 331 are parsimony-informative. Maximum parsimony analysis yielded one equally parsimonious tree (TL = 814, CI = 0.8710, HI = 0.1290, RI = 0.8930, RC = 0.7778). The best model for the ITS dataset estimated and applied in the Bayesian analysis was GTR + I + G (lset nst = 6, rates = invgamma; prset statefreqpr = dirichlet (1,1,1,1). Bayesian analysis and ML analysis resulted in a similar topology to MP analysis with an average standard deviation of split frequencies = 0.009408 (BI). The phylogenetic tree indicated that *A. yunnanense* was grouped with the close taxa *A*. *cervicolor* (Berk. & M.A. Curtis) Massee.

The ITS dataset of the genus *Phanerochaete* (Figure 4) included sequences from 96 fungal specimens representing 60 species. The dataset had an aligned length of 880 characters, of which 319 characters are constant, 77 are variable and parsimony-uninformative, and 484 are parsimony-informative. Maximum parsimony analysis yielded one equally parsimonious tree (TL = 2187, CI = 0.4015, HI = 0.5985, RI = 0.6231, RC = 0.2501). The best model for the ITS dataset estimated and applied in the Bayesian analysis was GTR + I + G (lset nst = 6, rates = invgamma; prset statefreqpr = dirichlet (1,1,1,1). Bayesian analysis and ML analysis resulted in a similar topology to MP analysis with an average standard deviation of split frequencies = 0.001737 (BI). The phylogenetic tree indicated that *P. tongbiguanensis* was grouped with the close taxa *P*. *daliensis* J. Yu & C.L. Zhao.

The ITS dataset of the genus *Phlebiopsis* (Figure 5) included sequences from 33 fungal specimens representing 20 species. The dataset had an aligned length of 665 characters, of which 392 characters are constant, 82 are variable and parsimony-uninformative, and 191 are parsimony-informative. Maximum parsimony analysis yielded six equally parsimonious trees (TL = 685, CI = 0.5650, HI = 0.4350, RI = 0.6543, RC = 0.3697). The best model for the ITS dataset estimated and applied in the Bayesian analysis was GTR + I + G (lset nst = 6, rates = invgamma; prset statefreqpr = dirichlet (1,1,1,1). Bayesian analysis and ML analysis resulted in a similar topology to MP analysis with an average standard deviation of split frequencies = 0.003384 (BI). The phylogenetic tree indicated that *P. fissurata* was grouped with the close taxa *P*. *lamprocystidiata* (Sheng H. Wu) Sheng H. Wu & Hallenb.

The ITS dataset of the genus *Vararia* (Figure 6) included sequences from 52 fungal specimens representing 40 species. The dataset had an aligned length of 796 characters, of which 148 characters were constant, 116 were variable and parsimony-uninformative, and 532 were parsimony-informative. Maximum parsimony analysis yielded one equally parsimonious tree (TL = 4063, CI = 0.3104, HI = 0.6896, RI = 0.4313, and RC = 0.1339). The best model for the ITS dataset estimated and applied in the Bayesian analysis was GTR + I + G. The Bayesian and ML analyses resulted in a similar topology to that of the MP analysis with split frequencies = 0.000442 (BI). The phylogram inferred from ITS sequences (Figure 6) revealed that *V. yingjiangensis* was grouped with six close taxa, namely *V. ambigua* Boidin, Lanq. & Gilles, *V*. *ellipsospora* G. Cunn., *V*. *fragilis* L. Zou & C.L. Zhao, *V*. *gallica* (Bourdot & Galzin) Boidin, *V*. *ochroleuca* (Bourdot & Galzin) Donk and *V. tropica* A.L. Welden.

### 3.2. Taxonomy

**Figure 7:**
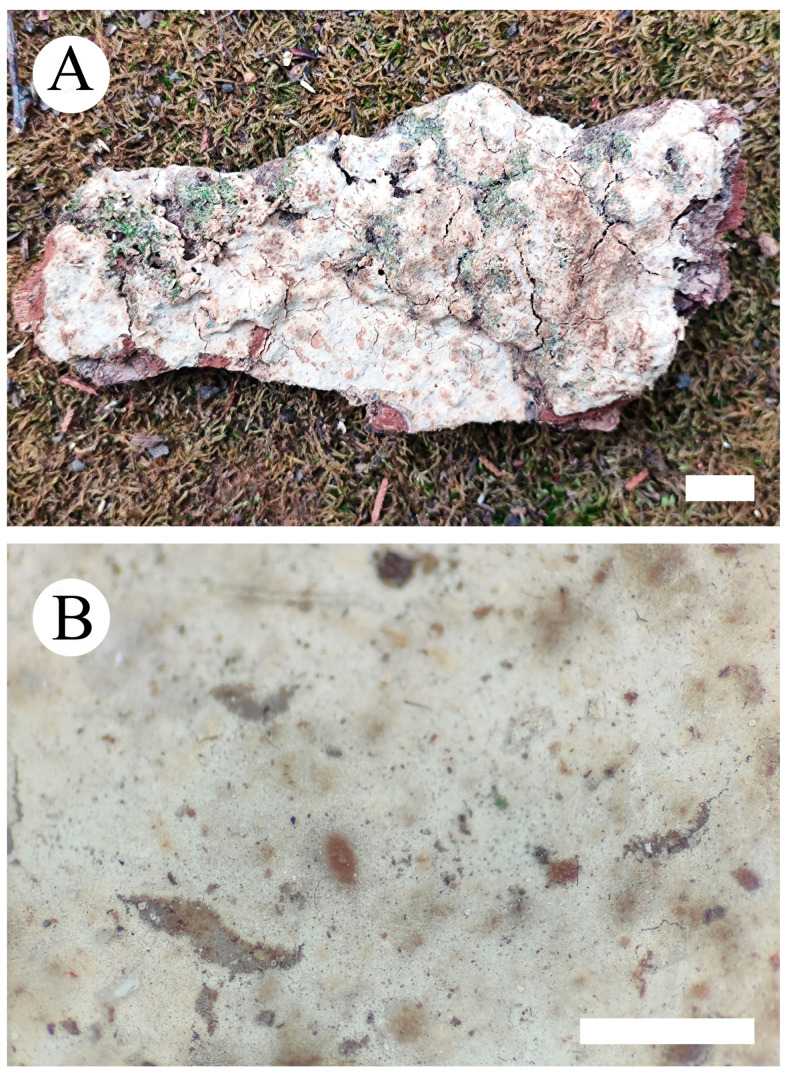
Basidiomata of *Asterostroma yunnanense* (holotype): the front of the basidiomata (**A**); characteristic hymenophore (**B**). Bars: (**A**) = 1 cm and (**B**) = 1 mm.

**Figure 8:**
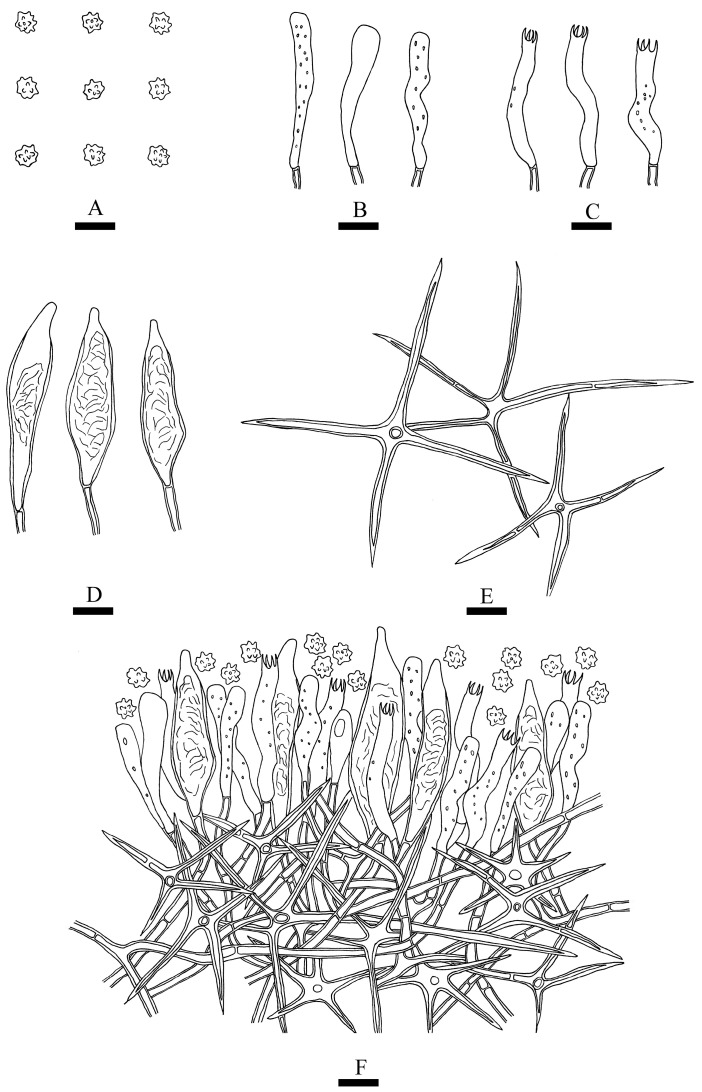
Microscopic structures of *Asterostroma yunnanense* (holotype): basidiospores (**A**), basidioles (**B**), basidia (**C**), gloeocystidia (**D**), asterosetae from subiculum (**E**), and a section of hymenium (**F**). Bars: (**A**–**F**) = 10 µm.

***Asterostroma yunnanense*** Y.L. Deng & C.L. Zhao, sp. nov. Figure 7 and Figure 8.

MycoBank no.: 851416

**Holotype**—China, Yunnan province, Lincang, Fengqing County, Yaojie Town, Xingyuan Village, 24°58′ N, 99°92′ E, altitude 1660 m asl., on the fallen branch of angiosperm, leg. C.L. Zhao, 20 July 2022, CLZhao 22781 (SWFC).

**Etymology**—***Yunnanense*** (Lat.): referring to the locality (Yunnan province) of the type specimen.

**Fruiting body**—Basidiomata annual, resupinate, membranaceous to pellicular, soft, without odor and taste when fresh, up to 110 mm long, 60 mm wide, and 280 µm thick. Hymenial surface smooth, cream when fresh, cream to salmon-buff, sometimes cracked when dried. Sterile margin thinning out, becoming indistinct and concolorous with hymenophore surface, up to 1 mm.

**Hyphal system**—Dimitic, generative hyphae bearing simple-septa, scattered, thick-walled, colorless, 2–4 µm in diameter, IKI-, CB-, tissues unchanged in KOH. Asterosetae in subiculum are abundant, predominant, yellowish brown, thick-walled, 2–4 µm in diameter, regularly star-shaped, weakly dextrinoid, rays up to 60 µm long, with acute tips, CB-, tissues unchanged in KOH.

**Hymenium**—Gloeocystidia subulate, thick-walled, with a basal simple septum, 34.5–54 × 7–10 µm. Basidia cylindrical, colorless, with four sterigmata and a basal simple-septum, 31–38 × 4–5 µm.

**Basidiospores**—Globose, colorless, thin-walled, echinulate, amyloid, 4.5–6 × 4–5 µm, L = 5.11 µm, W = 4.33 µm, Q = 1.07–1.18 (n = 60/2).

**Additional specimens examined (paratypes)**—China, Yunnan province, Lincang, Fengqing County, Yaojie Town, Xingyuan Village, 24°58′ N, 99°92′ E, altitude 1660 m asl., on the trunk of angiosperm, leg. C.L. Zhao, 20 July 2022, CLZhao 22786 (SWFC); on the fallen branch of angiosperm, leg. C.L. Zhao, 20 July 2022, CLZhao 22846 (SWFC).

**Figure 9:**
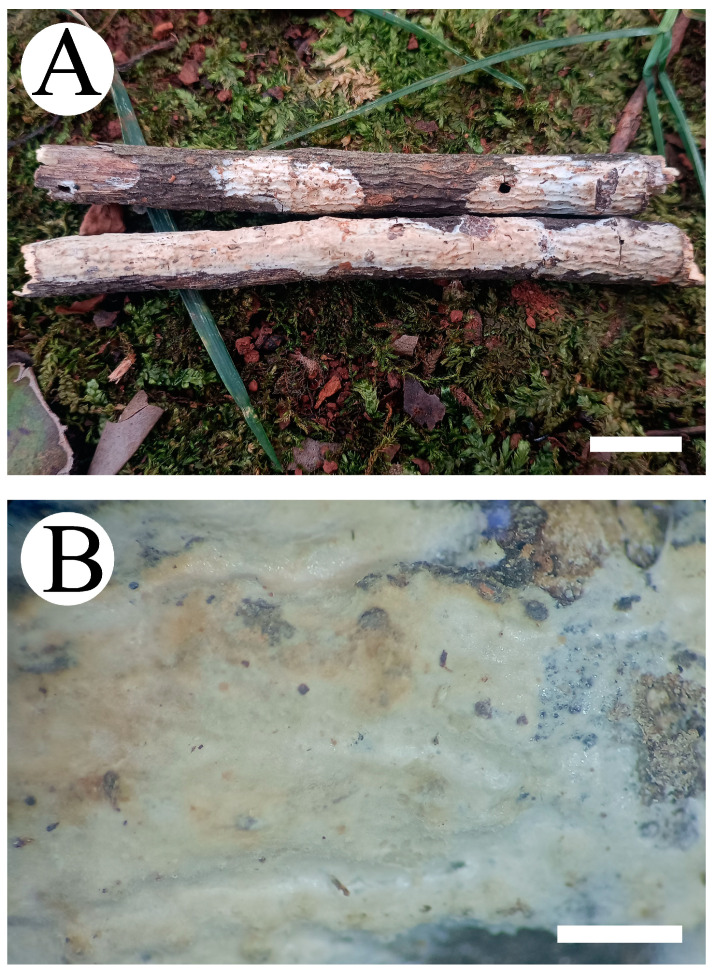
Basidiomata of *Phanerochaete tongbiguanensis* (holotype): the front of the basidiomata (**A**); characteristic hymenophore (**B**). Bars: (**A**) = 1 cm and (**B**) = 1 mm.

**Figure 10:**
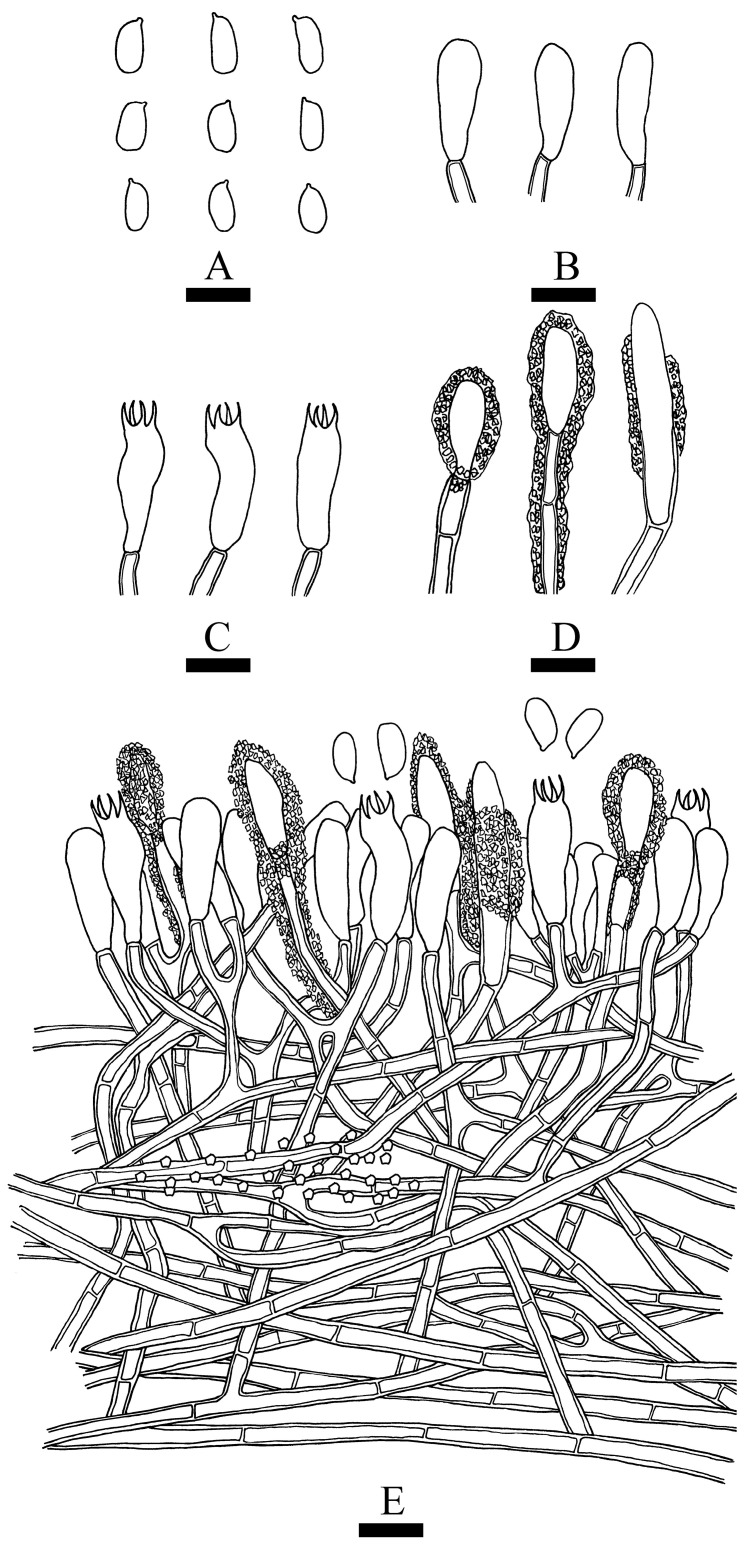
Microscopic structures of *Phanerochaete tongbiguanensis* (holotype): basidiospores (**A**), basidioles (**B**), basidia (**C**), cystidia (**D**), and A section of hymenium (**E**). Bars: (**A**–**E**) = 10 µm.

***Phanerochaete tongbiguanensis*** Y.L. Deng & C.L. Zhao sp. nov. Figure 9 and Figure 10.

MycoBank no.: 851417

**Holotype**—China, Yunnan province, Dehong, Yingjiang County, Tongbiguan Provincial Nature Reserve, 24°71′ N, 97°94′ E, altitude 2000 m asl., on the fallen branch of angiosperm, 20 July 2023, CLZhao 30606 (SWFC).

**Etymology**—***Tongbiguanensis*** (Lat.): referring to the locality (Tongbiguan) of the type specimen.

**Fruiting body**—Basidiomata annual, resupinate, thin, adnate, leather, without odor and taste when fresh, up to 70 mm long, 10 mm wide, 70–130 µm thick. Hymenial surfaces are smooth, white to cream when fresh, to cream to slightly buff upon drying. Sterile margins are distinct, whitish, and up to 1 mm.

**Hyphal system**—Monomitic, generative hyphae bearing simple-septa, thick-walled, 3–4 µm in diameter, branched, colorless, IKI-, CB-; tissues unchanged in KOH; subhymenial hyphae densely covered by crystals.

**Hymenium**—Cystidia subclavate, colorless, covered with a lot of crystals, thick-walled, 32–41 × 6.5–11 µm. Basidia subclavate to cylindrical, with four sterigmata and a basal simple septum, 17–26 × 6–7 µm.

**Basidiospores**—Oblong ellipsoid, colorless, thin-walled, smooth, IKI-, CB-, 6–9 × 3–4.5 µm, L = 7.48 µm, W = 4.02 µm, Q = 1.84–1.88 (n = 60/2).

**Additional specimen examined (paratype)**—China, Yunnan province, Dehong, Yingjiang County, Tongbiguan Provincial Nature Reserve, 24°71′ N, 97°94′ E, altitude 2000 m asl., on the fallen branch of angiosperm, 20 July 2023, CLZhao 30628 (SWFC).

**Figure 11:**
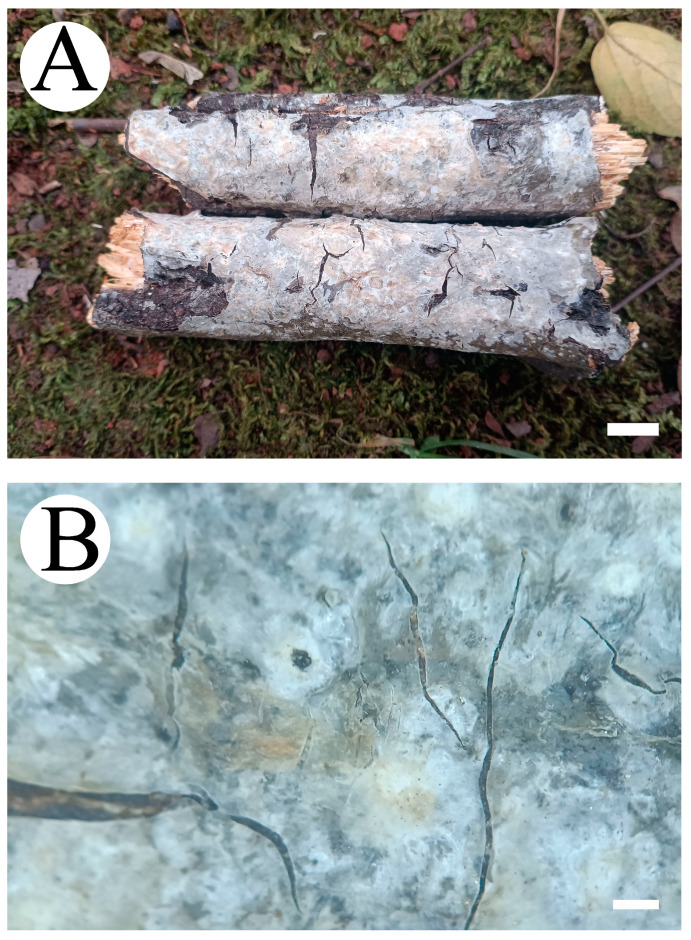
Basidiomata of *Phlebiopsis fissurata* (holotype): the front of the basidiomata (**A**); characteristic hymenophore (**B**). Bars: (**A**) = 1 cm and (**B**) = 1 mm.

**Figure 12:**
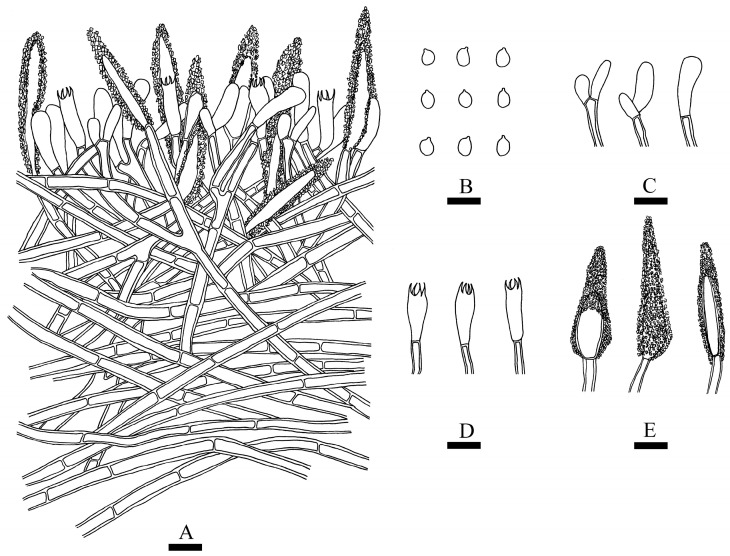
Microscopic structures of *Phlebiopsis fissurata* (holotype): a section of hymenium (**A**), basidiospores (**B**), basidioles (**C**), basidia (**D**), and cystidia (**E**). Bars: (**A**–**E**) = 10 µm.

***Phlebiopsis fissurata*** Y.L. Deng & C.L. Zhao sp. nov. Figure 11 and Figure 12.

MycoBank: 851421

**Holotype**—China, Yunnan province, Dehong, Yingjiang County, Tongbiguan Provincial Nature Reserve, 24°71′ N, 97°94′ E, altitude 2000 m asl., on the fallen branch of angiosperm, 19 July 2023, CLZhao 30147 (SWFC).

**Etymology**—Referring to the cracking hymenial surface.

**Fruiting body**—Basidiomata annual, resupinate, adnate, membranaceous, without odor and taste when fresh, up to 100 mm long, 70 mm wide, 100–210 µm thick. Hymenial surface tuberculate, white when fresh, white to buff to slightly brown upon drying, sometimes sparsely and deeply cracked with age. Sterile margins are distinct, white, and up to 2 mm.

**Hyphal system**—Monomitic, generative hyphae bearing simple-septa, colorless, thick-walled, branched, interwoven, 4–5 µm in diameter, IKI-, CB-; tissues unchanged in KOH.

**Hymenium**—Cystidia conical, colorless, covered with a lot of crystals, thick-walled, 27–48 × 6–11 µm. Basidia clavate, with four sterigmata and a basal simple septum, 16–26 × 5–7 µm.

**Basidiospores**—Broadly ellipsoid, thin-walled, colorless, smooth, IKI-, CB-, 4–6.5 × 3–4 µm, L = 5.03 µm, W = 3.59 µm, Q = 1.33–1.47 (n = 60/2).

**Additional specimen examined (paratype)**—China, Yunnan province, Dehong, Yingjiang County, Tongbiguan Provincial Nature Reserve, 24°71′ N, 97°94′ E, altitude 2000 m asl., on the fallen branch of angiosperm, 19 July 2023, CLZhao 30247 (SWFC).

**Figure 13:**
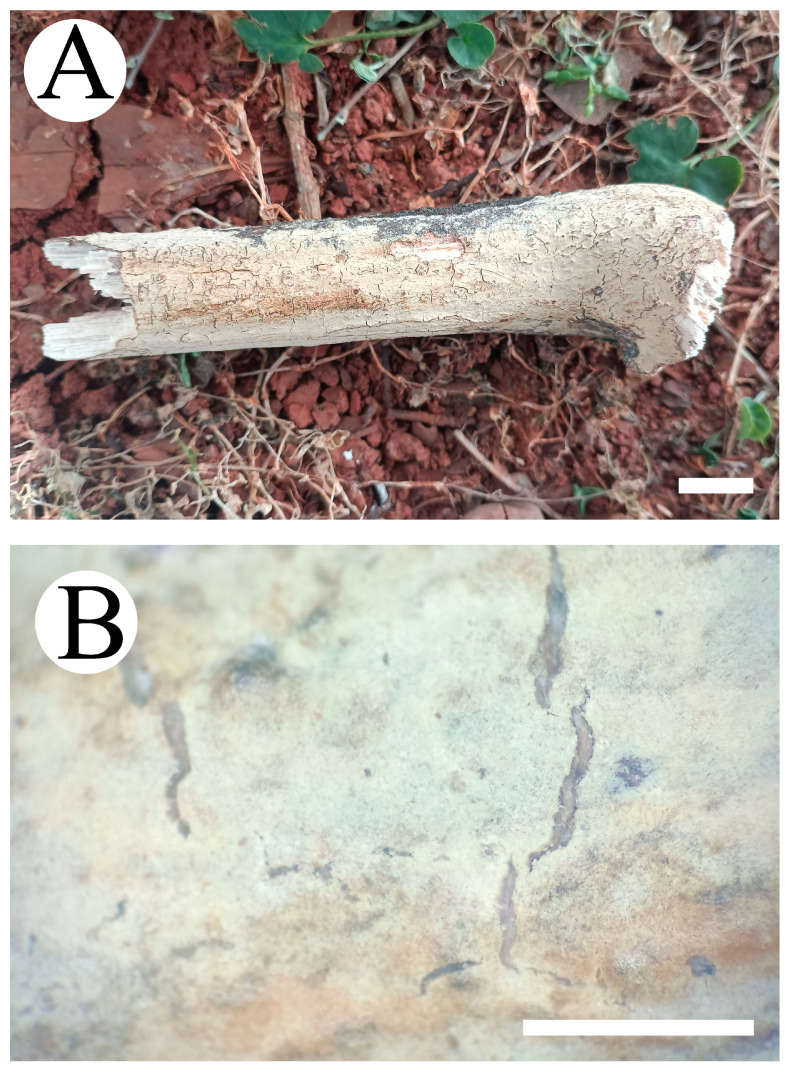
Basidiomata of *Vararia yingjiangensis* (holotype): the front of the basidiomata (**A**); characteristic hymenophore (**B**). Bars: (**A**) = 1 cm and (**B**) = 1 mm.

**Figure 14:**
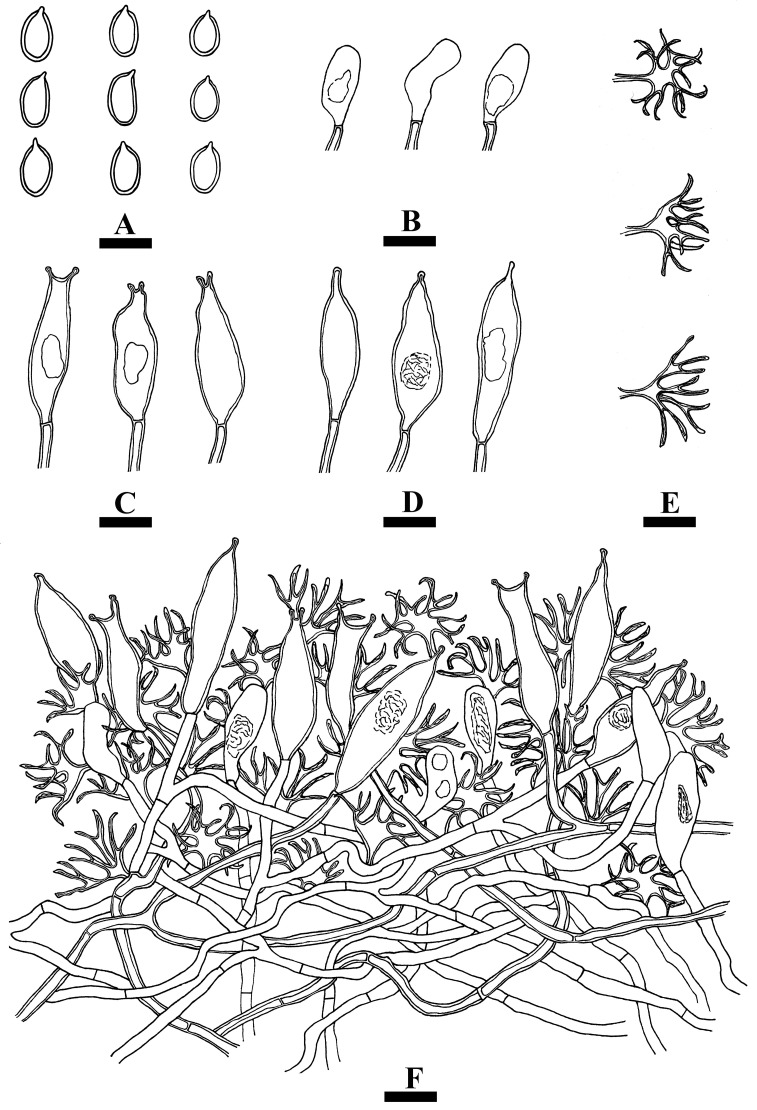
Microscopic structures of *Vararia yingjiangensis* (holotype): basidiospores (**A**), basidioles (**B**), gloeocystidia (**C**,**D**), dichohyphae (**E**), and a section of hymenium (**F**). Bars: (**A**–**F**) = 10 µm.

***Vararia yingjiangensis*** Y.L. Deng & C.L. Zhao sp. nov. Figure 13 and Figure 14.

MycoBank no.: 851424

**Holotype**—China, Yunnan province, Dehong, Yingjiang County, Tongbiguan Provincial Nature Reserve, 24°71′ N, 94°52′ E, altitude 1500 m asl., on fallen branch of angiosperm, 19 July 2023, CLZhao 30284 (SWFC).

**Etymology**—***Yingjiangensis*** (Lat.): referring to the locality (Yingjiang) of the type specimen.

**Fruiting body**—Basidiomata annual, adnate, corky, without odor and taste when fresh, up to 80 mm long, 40 mm wide, 80–120 µm thick. Hymenial surface smooth, cream to pinkish buff when fresh, pinkish buff to cinnamon-buff when dry, cracking with age. Sterile margin thin, indistinct, slightly cream to pinkish buff, up to 2 mm.

**Hyphal system**—Dimitic, generative hyphae bearing simple-septa, colorless, thin- to thick-walled, occasionally branched, interwoven, 3–4 µm in diameter, IKI-, CB-, tissues unchanged in KOH. Dichohyphae yellowish, capillary, distinctly thick-walled, up to 1.4 μm in diameter and with acute tips, moderately dextrinoid in Melzer’s reagent; more frequently branched.

**Hymenium**—Gloeocystidia two types, (i) Gloeocystidia subulate, usually with a constriction at the tip, colorless, obviously thick-walled, smooth, 25–42.5 × 5–11 µm; (ii) Gloeocystidia subulate, usually with two constrictions at the tip, colorless, obviously thick-walled, smooth, 28–35 × 6–10 µm. Basidia rare; basidioles cylindrical, dominant, thin-walled, 13–26 × 4.5–10 µm.

**Basidiospores**—Ellipsoid, slightly thick-walled, colorless, smooth, amyloid, CB-, 6.5–11.5 × 5–7 µm, L = 9.34 µm, W = 6.08 µm, Q = 1.5–1.6 (n = 60/2).

**Additional specimen examined (paratype)**—China, Yunnan province, Dehong, Yingjiang County, Tongbiguan Provincial Nature Reserve, 24°71′ N, 97°52′ E, altitude 1500 m asl., on fallen branch of angiosperm, 19 July 2023, CLZhao 30392 (SWFC).

## 4. Discussion

The family-level classification for the order Polyporales (Basidiomycota) revealed that the two taxa of *Phanerochaete daliensis* and *Phlebiopsis lamprocystidiata* nested into the family Phanerochaetaceae within the residual polyporoid clade based on the molecular systematics study amplifying the ITS, nLSU, RPB1, and RPB2 genes [21,27]. Seven genera, *Asterostroma*, *Dichostereum*, *Gloiothele*, *Peniophora*, *Scytinostroma*, *Vararia*, and *Vesiculomyces* E. Hagstr., were grouped together and clustered within the family Peniophoraceae [18]. In the present study, four new species were nested into the families Phanerochaetaceae and Peniophoraceae; from the phylogram of the ITS + nLSU data, the new species *Phanerochaete tongbiguanensis* were grouped into *Phanerochaete*, and the taxon *Phlebiopsis fissurata* was grouped into genus *Phlebiopsis* (Figure 1)*;* the new species *Asterostroma yunnanense* was grouped into *Asterostroma*, and *Vararia yingjiangensis* was clustered into *Vararia* (Figure 2).

Based on ITS topology (Figure 3), the present study revealed that the new species *Asterostroma yunnanense* was grouped with two close taxa, *A. cervicolor* and *A. vararioides* S.L. Liu & S.H. He. However, morphologically, *A. cervicolor* is distinct from *A. yunnanense* by the thin-walled marginal hyphae (2–5 µm diameter) and thin-walled aerial hyphae 1–5 µm diameter, and smaller gloeocystidia (20–30 × 7–15 µm) [86]. The species *A. vararioides* can be distinguished by its grayish brown to dark brown hymenial surface, thin-walled generative hyphae, presence of the dichohyphidia and thin-walled, longer gloeocystidia (30–60 × 5–11 µm), larger subcylindrical to fusoid basidia (30–65 × 7–11 µm), and larger, smooth basidiospores measuring 5.5–7.5 × 5–7 µm [18].

The phylogenetic tree (Figure 4) based on the ITS data showed that the new taxon *P. tongbiguanensis* was grouped with the species *P. daliensis* and *P. subtropica* J. Yu & C.L. Zhao. However, morphologically, *P. daliensis* is distinct from *P. tongbiguanensis* by its grandinioid hymenophore, ellipsoid e to cylindrical, thick-walled, smaller basidiospores (3–6 × 1.8–3 µm) [27]. The species *P. subtropica* is distinguished from *P. tongbiguanensis* by its fusiform cystidia and smaller basidia (12–21 × 3–5 µm) and ellipsoid basidiospores measuring as 3.0–4.8 × 2.4–3.4 µm [27]. Phylogenetic tree analysis (Figure 5) revealed that the new species *P. fissurata* was grouped with the species *P. lamprocystidiata* and then closely clustered with *P. yunnanensis* C.L. Zhao and *P. gigantea* (Fr.) Jülich. However, morphologically, *P. lamprocystidiata* is distinct from *P. fissurata* by its grayish yellow hymenial surface and distinct lamprocystidia [31]. The taxon *P. yunnanensis* is distinct from *P. fissurata* by having the smaller, narrowly clavate to subcylindrical basidia (10–21 × 3.5–4.5 µm) and smaller basidiospores measuring as 3.5–4.5 × 2.5–3.5 µm. Another species *P. gigantea* can be distinguished by its greyish-white to buff basidimata, larger cystidia (50–80 × 10–15 µm), and narrowly ellipsoid, smaller basidiospores (6.5–8 × 3–3.5 µm) [13].

Based on the ITS phylogenetic analysis (Figure 6), the new species *Vararia yingjiangensis* is closely grouped with six taxa, namely *V. ambigua*, *V. ellipsospora*, *V. fragilis*, *V. gallica*, *V. ochroleuca*, and *V. tropica*. However, morphologically, *V. ochroleuca* is distinct from *V. yingjiangensis* by having the slightly thick-walled gloeocystidia, thin-walled generative hyphae, and both smaller gloeocystidia (16–34 × 4.5–7.5 µm) and basidiospores measuring as 2.6–3.8 × 2–3.2 µm [87]. The taxon *V. gallica* is distinct from *V. yingjiangensis* by having the longer basidiospores measuring as 9–12 × 3.5–5 µm [24]. The species *V. ellipsospora* is distinct from *V. yingjiangensis* by having the fimbriate basidiomata, generative hyphae with clamped connection, and flexuous to cylindrical gloeocystidia [45]. *Vararia fragilis* differs from *V. yingjiangensis* by having smaller, elliptical to ovoid gloeocystidia measuring as 5.8–16 × 3.5–7 µm. The taxon *V. ambigua* differs from *V. yingjiangensis* by its thin-walled and smaller spores measuring as 3–8 × 3–5 µm. The species *V. tropica* can be distinguished by its wider, oblong basidiospores (10–12 × 7–8 µm) [88].

Based on the phylogenetic and morphological research results, more and more new wood-inhabiting fungi are being found and reported [1,43,54,55,89,90,91,92]. In the present study, four new taxa from the subtropics are described based on morphological and molecular phylogenetic analyses, which can enrich the wood-inhabiting fungal diversity in China and the world.

## Figures and Tables

**Table 1:** PCR reaction system and reaction conditions.

Genes	Primers	Temperature	Time	
ITS	Primer (10 µmol/L) (ITS 5) Primer (10 µmol/L) (ITS 4)	Predegeneration 94 °C	1.5 min	35 cycles
		Denaturation 94 °C	30 s	
		Renaturation 55 °C	45 s	
		Extend 72 °C	1 min	
		Extend 72 °C	10 min	
		Save 4 °C	—	
nLSU	Primer (10 µmol/L) (LROR) Primer (10 µmol/L) (LR 7)	Predegeneration 94 °C	1.5 min	
		Denaturation 94 °C	20 s	
		Renaturation 48 °C	1.5 min	
		Extend 72 °C	1.5 min	
		Extend 72 °C	5 min	
		Save 4 °C	—	

## Data Availability

Publicly available datasets were analyzed in this study.
